# Combined effect of triglyceride-glucose index and glucose disposal rate on cardio-cerebrovascular disease

**DOI:** 10.1371/journal.pone.0342154

**Published:** 2026-02-06

**Authors:** Hongfei Yang, Chao Sun, Ya Li, You Zhou, Rui Wang, Yingxue Li

**Affiliations:** Department of Neurology, Yingshang County People’s Hospital, Fuyang, Anhui, China; University of Diyala College of Medicine, IRAQ

## Abstract

**Objective:**

The triglyceride-glucose index and estimated glucose disposal rate serve as notable surrogate markers of insulin resistance, demonstrating established links to cardio-cerebrovascular disease. However, their combined prognostic value in predicting cardio-cerebrovascular disease outcomes remains unexplored. The current investigation examined the interaction between the TyG (triglyceride–glucose index) index and eGDR (estimated glucose disposal rate) concerning the danger of cardiovascular disease within a clinical population.

**Methods:**

This investigation employed data sourced from the China Health and Retirement Longitudinal Study (CHARLS). The median TyG index and eGDR scores were used to stratify the participants into 4 categories: low TyG/high eGDR, high TyG/high eGDR, low TyG/low eGDR, and high TyG/low eGDR. Clinical characteristics across groups were systematically compared. Cox proportional hazards regression models evaluated the distinct and interconnected associations of the TyG index and eGDR with the risk of cardio-cerebrovascular disease, with multiplicative and additive interaction effects subsequently assessed through formal interaction analysis.

**Results:**

The final study cohort comprised 7,330 participants, with 1,336 individuals (18.2%) developing cardio-cerebrovascular disease during the 9-year follow-up. Stratification using median thresholds (TyG: 8.59; eGDR: 10.55 mg/kg/min) yielded four groups: low TyG/high eGDR (n = 2,991), high TyG/high eGDR (n = 1,375), low TyG/low eGDR (n = 1,372), and high TyG/low eGDR (n = 2,292). Multivariable-adjusted Cox regression analyses revealed markedly increased risks of cardio-cerebrovascular disease among the various exposure groups when contrasted with the low TyG/high eGDR reference: high TyG/high eGDR (HR: 1.31, 95%CI: 1.10–1.57, *p*< 0.05), low TyG/low eGDR (HR: 1.54, 95%CI: 1.29–1.84, *p*< 0.05), and high TyG/low eGDR (HR: 1.55, 95%CI: 1.31–1.82, *p*< 0.05). Interaction analysis revealed significant multiplicative effects between TyG and eGDR but no evidence of additive interaction.

**Conclusion:**

The TyG index and eGDR demonstrate independent associations with cardio-cerebrovascular disease risk, while their combined assessment reveals synergistic predictive capacity. Combined assessment of the two allows for further accurate stratification of the population based on insulin resistance and improved prediction of cardio-cerebrovascular disease.

## 1. Introduction

Cardio-cerebrovascular disease (CCVD), encompassing coronary heart disease (CHD), cerebrovascular incidents, cardiac insufficiency, and associated conditions of the cardiovascular system [1], remains a leading global health burden. Worldwide CCVD prevalence reached 523.2 million cases in 2019 [2], with China accounting for approximately 330 million affected individuals in 2023, comprising 13 million occurrences of stroke, 11.39 million individuals diagnosed with CHD, and 8.9 million cases of cardiovascular disease [3]. As per the 2022 China Health Statistics Yearbook [4], CCVD constitutes the foremost contributor to mortality rates in cities as well as rural demographics, representing over 40% of total disease-related deaths. These epidemiological trends underscore the critical need for identifying modifiable risk factors, implementing early risk stratification, and developing targeted prevention strategies for cardio-cerebrovascular disease.

Insulin resistance (IR) is defined by a diminished physiological reaction of insulin-responsive tissues to hormonal stimuli [5], adversely affects glucose metabolism in hepatic, muscular, and adipose systems. This metabolic dysfunction constitutes a key pathophysiological mechanism underlying cardio-cerebrovascular disease disorders [6–8]. Early identification of IR-prone individuals, coupled with therapeutic interventions to restore insulin sensitivity, may effectively mitigate disease progression and enhance clinical outcomes in cardio-cerebrovascular disease prevention strategies.

The hyperinsulinemic-euglycemic clamp approach continues to be seen as the benchmark for evaluating IR, though its clinical application remains limited by procedural complexity and resource intensity [9]. In practice, surrogate biomarkers such as the TyG index and eGDR have emerged as reliable alternatives for insulin resistance evaluation. These calculated indices, derived from routine metabolic parameters, enable cost-effective risk stratification in both clinical and epidemiological research contexts.

While the TyG index and eGDR have been independently investigated for their associations with cardio-cerebrovascular disease [10–12], evidence regarding their combined prognostic utility remains scarce. This investigation rigorously investigates the synergistic connection among TyG and eGDR in forecasting cardio-cerebrovascular disease outcomes via thorough interaction analyses.

## 2. Resources and techniques

Informed consent was obtained from every person involved, and the protocol received approval from the Ethical Review Board of Peking University (IRB00001052–11015). The research meticulously followed the guidelines established in the Declaration of Helsinki.

### 2.1. Population of study and sources of data

This research employed data derived from the CHARLS, a comprehensive longitudinal survey that constitutes a nationwide representative, targeting Chinese individuals from 45 years and above from randomly chosen households [13]. Initiated in the year 2011 with a cohort of 17,708 individuals spanning 150 counties and districts across 28 provinces, CHARLS employs biennial/biennial follow-ups through computer-assisted personal interviews (CAPI) to collect comprehensive demographic, socioeconomic, health status, and healthcare utilization data. Our analysis incorporated five survey waves (2011、2013、2015、2018、2020) to ensure longitudinal consistency and temporal validity.

**Fig 1:**
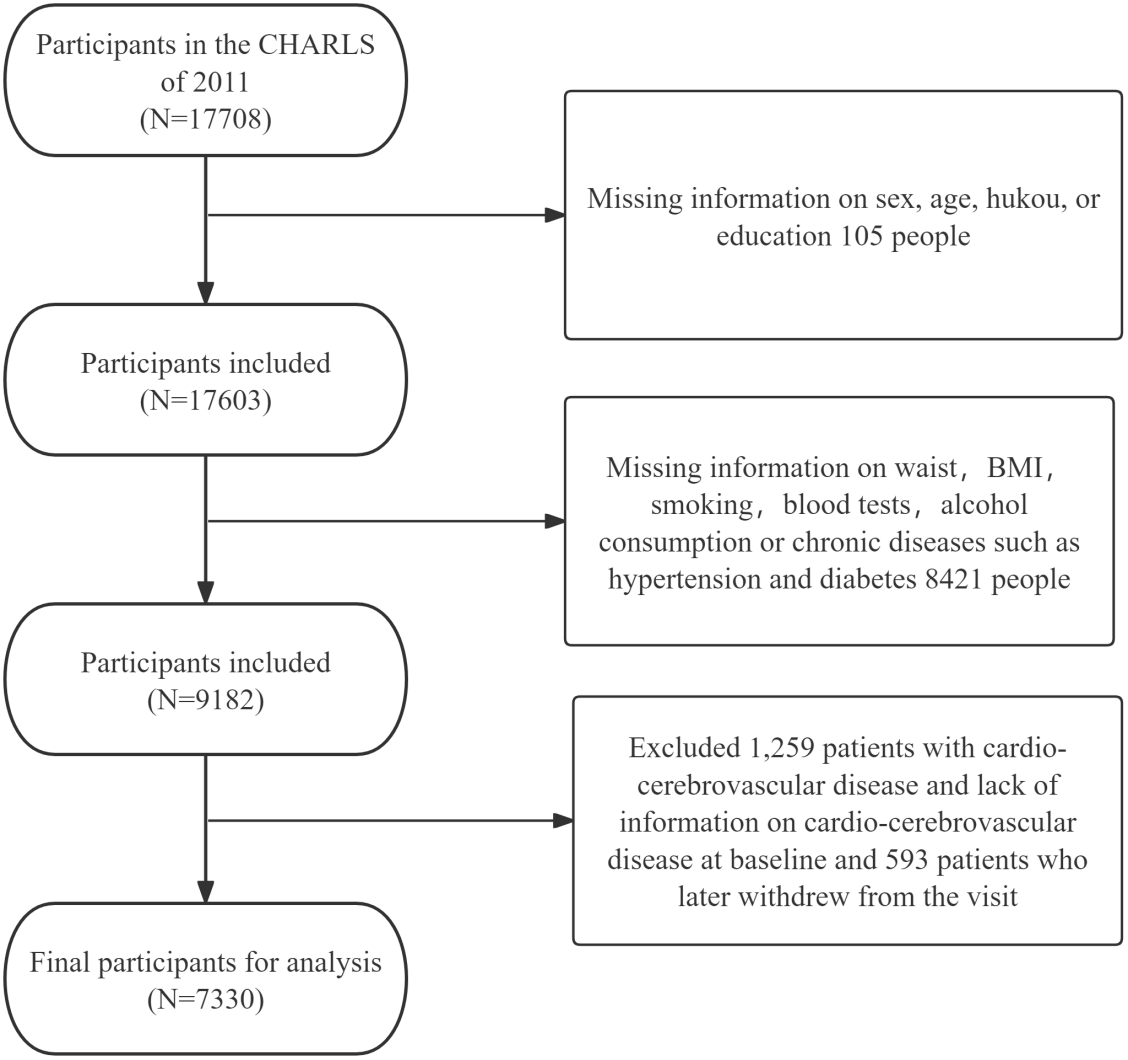
Participant selection process flowchart.

Initial cohort comprised 17,708 Wave 1 participants. Exclusion criteria included: (1) pre-existing cardio-cerebrovascular disease (CCVD) at baseline, (2) incomplete CCVD status documentation, (3) missing baseline covariates or biochemical data, and (4) loss to follow-up. Fig 1 depicts the model for picking subjects.

### 2.2. TyG index and eGDR assessment

The formulas for TyG index and eGDR are as below: (1) TyG index = In [fasting triglycerides (mg/dl) × fasting blood glucose (mg/dl)/2];(2) eGDR = 21.158 – (0.09 × waist circumference [cm]) – (3.407 × hypertension [yes 1 or no 0]) -(0.551 × glycosylated hemoglobin [%]) [14].

Levels of blood glucose, triglycerides, and hemoglobin A1c (HbA1c) were assessed following an overnight fasting period (≥8 hours).

### 2.3. Definition of CCVD

The current investigation focused on CCVD as the primary outcome variable of concern, which encompasses diseases related to cardiovascular conditions and cerebrovascular disorders. The evaluation of CCVD disease was conducted through responses to a series of inquiries in a follow-up survey [15]: “Has your doctor ever diagnosed you with heart disease, angina, coronary heart disease, heart failure, or other heart problems?” and “Has your doctor ever diagnosed you with a stroke?” Persons with a documented history of cardiovascular illness or stroke were defined as suffering from CCVD. Incident cardio-cerebrovascular disease events were analyzed as first incident occurrences, with simultaneous cardiovascular disease and cerebrovascular diagnoses within the same follow-up interval counted as a single event (index date corresponding to earliest diagnosis), ensuring non-repetitive outcome measurements regardless of disease co-occurrence.

### 2.4. Covariates

This study incorporated covariates assessed through baseline interviews, including demographic characteristics, health status indicators, and biochemical parameters. Demographic variables comprised sex (male/female), age, household registration type (agricultural/non-agricultural and other), and Level of education (low level: illiteracy, incomplete elementary school and graduation, private school; medium level: middle, high, and secondary school graduation; high level: college graduation, bachelor’s, master’s, and PhD graduation. Health-related metrics encompassed BMI (kg/m²), present cigarette usage status (yes/no), alcohol intake frequency (non-drinker/occasional [<1 drink monthly]/regular [≥1 drink monthly]), and physician-diagnosed comorbidities (hypertension, diabetes mellitus, dyslipidemia, cancer). The biochemical parameters encompassed fasting blood glucose (FBG), total cholesterol (TC), high-density lipoprotein cholesterol (HDL-C), low-density lipoprotein cholesterol (LDL-C), triglycerides (TG), blood urea nitrogen (BUN), serum creatinine (SCR), uric acid (UA), glycated hemoglobin (HbA1c), and platelet count (PLT).

### 2.5. Data analysis

Analysis of data was carried out utilizing SPSS 26.0 & R 4.4.2. In the statistical description phase, normality tests were conducted for continuous variables: continuous variables exhibiting a normal distribution are articulated as the means ± standard deviations (X ± S), while intergroup comparisons were performed via t tests. Nonnormally distributed continuous variables are expressed as medians (P_25_, P_75_), and the Mann‒Whitney U test was applied. Categorical variables are articulated through percentages and frequencies, while ratio analogies are executed utilizing the Fisher’s exact or chi-square test. Subjects got categorized into four distinct groups derived from the median thresholds of TyG and eGDR: low TyG/high eGDR, high TyG/high eGDR, low TyG/low eGDR, and high TyG/low eGDR.

During the statistical analysis phase, Kaplan-Meier analysis estimated cumulative cardio-cerebrovascular disease/cerebrovascular risk across TyG, eGDR, and combined indicator subgroups. Multivariable Cox proportional hazards regression models assessed connections through three sequential adjustments: Model 1 is not adjusted, Model 2 is adjusted for sex, age, type of household registration, and level of education, and Model 3 is Model 2 plus BMI, smoking, drinking, high blood pressure, diabetes, dyslipidemia, and cancer. A *p* value less than 0.05 was deemed of statistical importance. Finally, an interaction analysis was conducted, identifying high levels of TyG and low levels of eGDR as precursors for CCVDs. Interaction analysis between TyG index and eGDR involved two components: 1) Multiplicative interaction assessment through Cox regression modeling; 2) Additive interaction evaluation using Relative Excess Risk Due to Interaction (RERI), attributable proportion of interaction (AP), and synergy index (SI), interpretation of additive interaction significance based on established criteria (RERI > 0, AP > 0, or SI > 1) [16]. The level of importance was determined at *p*< 0.05 for all interaction evaluations.

## 3. Outcomes

### 3.1. General information

The study cohort comprised 7,330 participants (46.1% male, 53.9% female) with a median age of 58 (51–64)years. Population median values established stratification thresholds: TyG index 8.59 (low: < 8.59, high: ≥ 8.59) and eGDR 10.55 mg/kg/min (low: < 10.55, high: ≥ 10.55).

The study population was stratified into four subgroups by TyG/eGDR levels: low TyG/high eGDR (n = 2,291, 31.2%), high TyG/high eGDR (n = 1,375, 18.8%), low TyG/low eGDR (n = 1,372, 18.7%), and high TyG/low eGDR (n = 2,292, 31.3%). Comparative analyses between groups demonstrated notable disparities (*p*< 0.05)in sociodemographic characteristics (gender, age, registration of households, level of education), aspects of lifestyle, anthropometric measures (BMI), and cardiometabolic comorbidities (hypertension, diabetes, dyslipidemia). Biochemical profiles also differed significantly across groups (*p*< 0.05) for BMI, FBG, total cholesterol, HDL-C, LDL-C, TyG, blood urea nitrogen, uric acid, and HbA1c levels. Table 1 displays the complete baseline features.

**Table 1:** Comparison of baseline information for TyG index and eGDR combined subgroups.

Variables	Total(n = 7330)	Low TyG and high eGDR (n = 2291)	High TyG and high eGDR (n = 1375)	Low TyG and low eGDR (n = 1372)	High TyG and low eGDR (n = 2292)	*t/Z/χ²* value	*p* value
**Gender**						67.314	<0.05
Male	3376(46.1%)	1210(52.8%)	595(43.3%)	624(45.5%)	947(41.3%)		
Female	3954(53.9%)	1081(47.2%)	780(56.7%)	748(54.5%)	1345(58.7%)		
**Age/(year)**	58.00(51.00, 64.00)	57.00(50.00, 64.00)	57.00(51.00, 64.00)	58.50(51.00, 65.00)	58.00(52.00, 64.00)	26.502	<0.05
**Hukou**						95.436	<0.05
Agricultural hukou	6279(85.7%)	2061(90.0%)	1221(88.8%)	1146(83.5%)	1851(80.8%)		
Nonagricultural hukou	1051(14.3%)	230(10.0%)	154(11.2%)	226(16.5%)	441(19.2%)		
**education level**						13.830	<0.05
Low level	5147(70.2%)	1629(71.1%)	999(72.7%)	965(70.3%)	1554(67.8%)		
Medium level	2104(28.7%)	642(28.0%)	363(26.4%)	387(28.2%)	712(31.1%)		
High level	79(1.1%)	20(0.9%)	13(0.9%)	20(1.5%)	26(1.1%)		
**BMI/(kg/m**^**2**^)	23.05(20.85, 25.64)	21.09(19.53, 22.70)	21.66(20.14, 23.27)	24.48(22.56, 26.70)	25.70(23.61, 27.83)	2589.281	<0.05
**Smoking**						48.999	<0.05
Yes	2817(38.4%)	1003(43.8%)	531(38.6%)	506(36.9%)	777(33.9%)		
No	4513(61.6%)	1288(56.2%)	844(61.4%)	866(63.1%)	1515(66.1%)		
**Drinking**						37.040	<0.05
Yes	1898(25.9%)	682(29.8%)	339(24.7%)	353(25.7%)	524(22.9%)		
a little	572(7.8%)	195(8.5%)	108(7.9%)	104(7.6%)	165(7.2%)		
No	4860(66.3%)	1414(61.7%)	928(67.5%)	915(66.7%)	1603(69.9%)		
**Hypertension**						1770.096	<0.05
Yes	1503(20.5%)	8(0.3%)	17(1.2%)	538(39.2%)	940(41.0%)		
No	5827(79.5%)	2283(99.7%)	1358(98.8%)	834(60.8%)	1352(59.0%)		
**Diabetes**						255.862	<0.05
Yes	336(4.6%)	24(1.0%)	21(1.5%)	59(4.3%)	232(10.1%)		
No	6994(95.4%)	2267(99.0%)	1354(98.5%)	1313(95.7%)	2060(89.9%)		
**Dyslipidemia**						176.728	<0.05
Yes	521(7.1%)	61(2.7%)	65(4.7%)	113(8.2%)	282(12.3%)		
No	6809(92.9%)	2230(97.3%)	1310(95.3%)	1259(91.8%)	2010(97.7%)		
**Cancer**						2.947	0.400
Yes	64(0.9%)	19(0.8%)	9(0.7%)	10(0.7%)	26(1.1%)		
No	7266(99.1%)	2272(99.2%)	1366(99.3%)	1362(99.3%)	2266(98.9%)		
**FBG/(mg/dl)**	102.24(94.32, 112.68)	96.66(93.60, 104.22)	104.94(97.202, 116.46)	98.82(92.21, 105.84)	109.98(100.80, 128.66)	1292.236	<0.05
**TC/(mg/dl)**	190.59(167.01, 214.95)	180.54(159.28, 204.51)	196.39(171.65, 222.68)	184.79(163.92, 205.67)	199.87(176.68, 226.93)	393.917	<0.05
**HDL-C/(mg/dl)**	49.87(40.98, 60.31)	57.60(49.48, 67.65)	46.78(38.27, 55.67)	52.96(44.85, 63.02)	42.53(35.57, 50.64)	1491.610	<0.05
**LDL-C/(mg/dl)**	114.05(93.17, 136.86)	107.86(89.30, 128.74)	115.21(92.78, 139.18)	115.21(97.04, 136.47)	118.88(95.20, 144.20)	105.485	<0.05
**TG/(mg/dl)**	103.54(74.34, 150.45)	72.57(58.41, 88.50)	143.37(121.25, 190.27)	77.88(63.72, 92.04)	157.53(124.79, 215.94)	4937.861	<0.05
**BUN/(mg/dl)**	15.15(12.55, 18.15)	15.35(12.63, 18.60)	14.99(12.38, 17.95)	15.34(12.73, 18.65)	14.85(12.44, 17.51)	26.349	<0.05
**SCR/(mg/dl)**	0.76(0.64, 0.88)	0.75(0.64, 0.86)	0.76(0.64, 0.87)	0.75(0.64, 0.87)	0.76(0.66, 0.89)	14.483	<0.05
**UA/(mg/dl)**	4.26(3.55, 5.11)	4.05(3.39, 4.88)	4.22(3.54, 5.05)	4.23(3.55, 5.01)	4.52(3.75, 5.43)	181.320	<0.05
**HBA1C/(%)**	5.10(4.90, 5.40)	5.00(4.80, 5.30)	5.00(4.80, 5.30)	5.20(4.90, 5.40)	5.30(5.00, 5.70)	660.500	<0.05
**PLT/(10** ^**9**^ **/L)**	207.00(162.00, 254.00)	205.00(161.00, 252.00)	209.00(164.00, 255.00)	204.00(159.00, 254.00)	209.00(162.00, 258.75)	5.726	0.126

FBG, fasting blood glucose; TC, total cholesterol; HDL-C, high-density lipoprotein cholesterol; LDL-C, low-density lipoprotein cholesterol; TG, triglycerides; BUN, blood urea nitrogen; SCR, serum creatinine; UA, uric acid; HbA1c, glycated hemoglobin; PLT, platelet.

### 3.2. The interplay of the TyG index and eGDR in the context of CCVD

**Fig 2:**
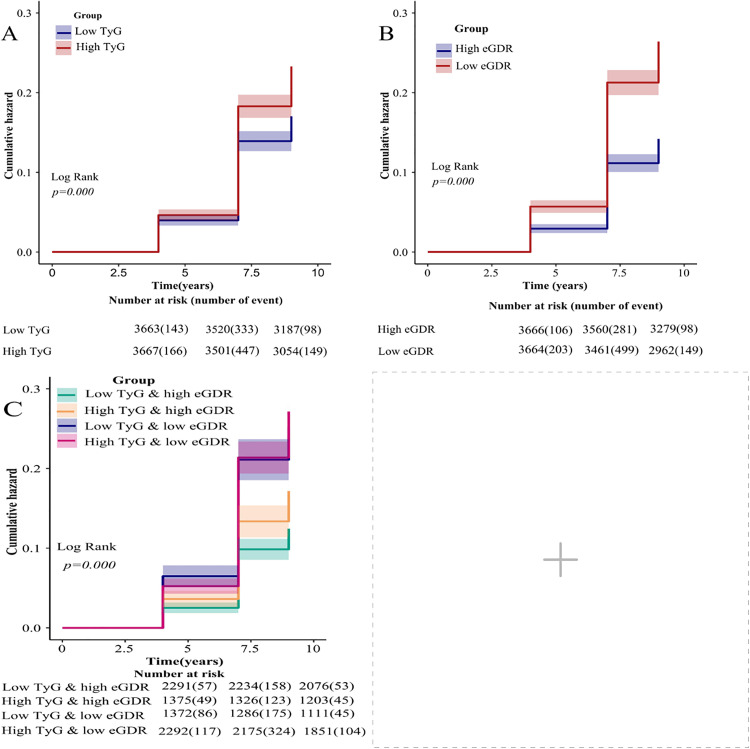
Kaplan-Meier Cumulative Risk Curves for TyG Index and eGDR and cardio-cerebrovascular disease. **(A)** TyG index grouping; **(B)** eGDR grouping; **(C)** Joint TyG index and eGDR grouping. Note: TyG: triglyceride-glucose; eGDR: glucose disposal rate.

Over a maximal follow-up period of nine years, 1336 subjects had cardio-cerebrovascular disease (961 cases of cardiovascular disease-related disease and 436 cases of cerebrovascular-related disease, both co-occurring in 61 cases during the same follow-up period, thus counting as 1336 cases of cardio-cerebrovascular disease).The prevalence rates in the four subgroups of the joint grouping of the TyG index and the eGDR were: low TyG and high eGDR (11.7% 268/2291), high TyG and high eGDR (15.8%, 217/1375), low TyG and low eGDR (22.3%, 306/1372), and high TyG and low eGDR (23.8%, 545/2292). The survival curves for Kaplan-Meier indicated that the overall incidence of cardio-cerebrovascular disease increased with increasing TyG and decreasing eGDR, and that in the integrated analysis of eGDR and TyG index, the incidence of the cumulative incidence of cardio-cerebrovascular disease was lowest in the low-TyG and high-eGDR groups and highest in the high-TyG and low-eGDR groups(*p*< 0.05). (Fig 2)

Multivariable-adjusted Cox regression analyses (Model 3) demonstrated that individuals exhibiting high levels of TyG showed a 1.16-fold increased cardio-cerebrovascular disease risk in contrast to individuals exhibiting reduced levels of TyG (HR: 1.16, 95%CI: 1.04–1.30, *p*< 0.05). Similarly, individuals with reduced eGDR showed a 1.38-fold higher risk relative to counterparts with higher eGDR values (HR: 1.38, 95%CI: 1.21–1.57, *p*< 0.05). When TyG index and eGDR were grouped jointly, using the low TyG and high eGDR group as a reference, the risk of high TyG and high eGDR cohort (HR: 1.31, 95%CI: 1.10–1.57, *p*< 0.05), low TyG and low eGDR (HR: 1.54, 95%CI: 1.29–1.84, *p*< 0.05), high TyG and low eGDR (HR: 1.55, 95%CI: 1.31–1.82, *p*< 0.05) had progressively higher risks. See Table 2.

**Table 2:** TyG index and eGDR and Cox proportional risk regression analysis of cardio-cerebrovascular disease.

Variables	Model 1		Model 2		Model 3	
**TyG**	HR (95%CI)	*p*	HR (95%CI)	*p*	HR (95%CI)	*p*
Low TyG	1.00 (Reference)		1.00 (Reference)		1.00 (Reference)	
High TyG	1.34 (1.20–1.50)	<0.05	1.30 (1.17–1.45)	<0.05	1.16 (1.04–1.30)	<0.05
**eGDR**						
High eGDR	1.00 (Reference)		1.00 (Reference)		1.00 (Reference)	
Low eGDR	1.82 (1.63–2.03)	<0.05	1.75 (1.56–1.95)	<0.05	1.38 (1.21–1.57)	<0.05
**TyG and** **eGDR**						
Low TyG and high eGDR	1.00 (Reference)		1.00 (Reference)		1.00 (Reference)	
High TyG and high eGDR	1.37 (1.14–1.64)	<0.05	1.34 (1.12–1.60)	<0.05	1.31 (1.10–1.57)	<0.05
Low TyG and low eGDR	1.99 (1.69–2.35)	<0.05	1.92 (1.63–2.30)	<0.05	1.54 (1.29–1.84)	<0.05
High TyG and low eGDR	2.11 (1.82–2.44)	<0.05	2.01 (1.73–2.32)	<0.05	1.55 (1.31–1.82)	<0.05

Note: Model 1: Uncorrected model; Model 2: Corrected for age, sex, hukou and level of literacy; Model 3: Corrected for BMI, cigarette and alcohol use, hypertension, diabetes mellitus, dyslipidemia, and cancer according to Model 2.

TyG: triglyceride-glucose; eGDR: glucose disposal rate.

### 3.3. The interplay of the TyG index and eGDR in relation to CCVD

**Fig 3:**
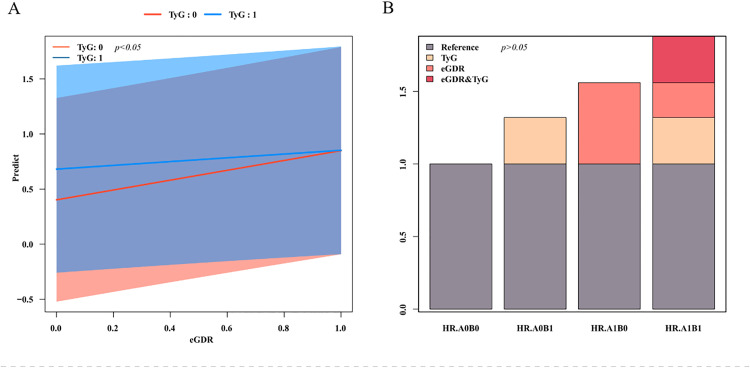
Interaction between TyG index and eGDR on cardio-cerebrovascular disease. **(A)** Plot of multiplicative interactions; **(B)** Plot of additive interactions. Note: TyG: triglyceride-glucose; eGDR: glucose disposal rate; TyG: 0: low TyG, TyG: 1: high TyG; AOBO: low TyG and high eGDR, AOB1: high TyG and high eGDR, A1B0: low TyG and low eGDR, A1B1: high TyG and low eGDR.

There was a notable multiplicative interplay of TyG and eGDR (OR: 0.76, 95%CI: 0.60–0.95, *p*< 0.05), suggests that their combined effect on CCVD risk is not a simple superimposition of their individual effects. However, the additive interaction was not statistically significant: RERI = −0.32 (95%CI: −0.64–0.01); AP = 0.20 (95%CI: −0.41–0.00); SI = 0.64 (95%CI: 0.44–0.94); *p*> 0.05. The outcomes suggest that in as much as TyG and eGDR interact on a multiplicative scale, they do not show a significant additive interaction (Table 3, Fig 3).

**Table 3:** Interplay of TyG index and eGDR on cardio-cerebrovascular disease.

Interactive indices	Model 1		Model 2		Model 3	
	Interactive effects(95%CI)	*p* value	Interactive effects(95%CI)	*p* value	Interactive effects(95%CI)	*p* value
Multiplicative effect	0.77 (0.61–0.97)	<0.05	0.78 (0.62–0.97)	<0.05	0.76 (0.60–0.95)	<0.05
Additive effect						
RERI	−0.25 (−0.63–0.13)	0.90	−0.26 (−0.62–0.11)	0.92	−0.32 (−0.64–0.01)	0.97
AP	−0.11 (−0.29–0.06)	0.10	−0.12 (−0.30–0.05)	0.08	−0.20 (−0.41–0.00)	<0.05
SI	0.82 (0.63–1.08)	0.40	0.81 (0.61–0.06)	0.41	0.64 (0.44–0.94)	0.46

Note: Model 1: Uncorrected model; Model 2: Corrected for gender, age, hukou and level of literacy; Model 3: Corrected for BMI, cigarette and alcohol use, hypertension, diabetes mellitus, dyslipidemia, and cancer according to Model 2.

## 4. Discussion

The hyperinsulinemic–euglycemic clamp (HEC), while continuing to serve as the benchmark for evaluating IR, faces clinical implementation barriers due to technical complexity. This has driven the adoption of practical surrogate measures. The TyG index, sourced through triglycerides and high-density lipoproteins, has gained popularity in evaluating IR and investigating associated diseases due to its straightforward measurement process and affordability. Guerrero-Romero et al. [17] demonstrated that the TyG index displayed a tight association with the HEC the premier technique for identifying IR. The threshold score for the TyG index (4.68) exhibited a sensitivity and specificity of 96.5% and 85% respectively in calculating IR among adults. Recent investigations have elucidated that the novel index eGDR, which is a straightforward measure incorporating waist size, high blood pressure, and glycosylated hemoglobin, shows a strong correlation with IR [18].

Recent developments highlight the importance of the TyG index as a notable indicator of CCVD risk among diverse groups. An NHANES (National Health and Nutrition Examination Survey) analysis of 6,502 older grown-ups (≥60 years) demonstrated a 30% increased cardiovascular disease risk per 1-unit TyG index increment (OR=1.30, 95%CI: 1.09–1.55, *p*= 0.003), with nonlinear risk escalation above the threshold TyG value of 8.73 [19]. In another study [20] suggested a more pronounced influence of TyG index on heart disease in young diabetic individuals, but two studies elucidated a weak association among TyG index and stroke. However, an investigation conducted by Rafiee H et al. [21] found that higher TyG related to cardiovascular disease (HR: 1.48, 95%CI: 1.22–1.79; *p*< 0.001; threshold = 8.91) and stroke (HR: 1.45, 95% CI: 0.96–2.19; *p*= 0.042; threshold = 8.92).. Consistently,Wang B et al. [22] also found an elevated risk of stroke due to high TyG index.

Although fewer studies have examined eGDR, the observed pattern is consistent: lower eGDR is associated with higher CCVD risk. A Sino-American longitudinal study of non-diabetic adults >50 years demonstrated an adverse linear association among overall eGDR and the event of CCVD (HR = 0.799 per unit increase, 95%CI: 0.726–0.878, *p*< 0.001) [23]. Central European population analyses similarly linked reduced eGDR levels with heightened cardio-cerebrovascular disease risk [12], and meta-analytic evidence indicates that low eGDR increases CCVD across type 1 and type 2 diabetes as well as in non-diabetic populations [24]. Together, TyG and eGDR appear to be clinically relevant IR markers with distinct risk profiles.In the current investigation, the Kaplan-Meier cumulative risk curves suggested that the elevated TyG cohort within the TyG classification exclusively and the diminished eGDR cohort within the eGDR classification exclusively elevated the incidence of cardio-cerebrovascular disease and cerebral vascular diseases, which aligns with earlier research findings. Evidence suggests that the TyG index, when used alongside other indices, may enhance the effectiveness of screening individuals at high danger for CCVD [22, 25, 26]. Importantly, two recent large-scale studies contextualize our joint findings. He et al. using NHANES 2001–2018, showed that high TyG + low eGDR conferred the highest hazards of all-cause and cardiovascular mortality, indicating an additive signal when the two indices are considered together [27]. Li et al. [28] analyzing NHANES and CHARLS, found that higher TyG and lower eGDR were each independently associated with current and future CVD, with eGDR demonstrating superior predictive performance. In line with these studies, we observed the lowest CCVD risk in the low-TyG/high-eGDR phenotype, followed by high-TyG/high-eGDR, low-TyG/low-eGDR, and high-TyG/low-eGDR, with hazard ratios of 1.31 (95%CI: 1.10–1.57), 1.54 (95%CI: 1.29–1.84), and 1.55 (95%CI 1.31–1.82), respectively (all *p*< 0.05). Notably, we detected an antagonistic interaction on the multiplicative scale (i.e., the incremental effect of TyG was attenuated when eGDR was already low), which may reflect ceiling/saturation of downstream pathways and overlapping biology captured by the two indices; differences in endpoint (incident CCVD vs. mortality) and modeling scale (multiplicative vs. additive) may also contribute to divergence from the additive signal in He et al.

Nonetheless, the existing literature on the interplay between the TyG index and eGDR is quite constrained. The investigation revealed that upon the simultaneous analysis of the TyG index and eGDR, the group with low TyG and high eGDR exhibited the least impact on CCVD. In comparison, the group characterized by elevated TyG and eGDR showed a hazard ratio of 1.31 (95%CI: 1.10–1.57, *p*< 0.05), whereas the low TyG and low eGDR group was also considered (HR: 1.54, 95%CI: 1.29–1.84, *p*< 0.05), and high TyG and low eGDR (HR: 1.55, 95%CI: 1.31–1.82, *p*< 0.05) had a gradually increasing risk. Findings indicate that the incorporation of the TyG index alongside eGDR may improve the segmentation of individuals based on IR, thereby refining the prediction of CCVD. The pathophysiological mechanisms linking insulin resistance to cardio-cerebrovascular disease involve multiple interconnected pathways:1.Chronic hyperglycemia promotes advanced glycation end-product (AGE) accumulation, inducing collagen deposition and myocardial fibrosis that impair cardiac function [29].2.Insulin resistance induces systemic inflammation and oxidative stress, damaging vascular endothelial integrity through reactive oxygen species-mediated pathways [30].3.Hypertriglyceridemia elevates circulating free fatty acids, increasing myocardial oxygen demand while reducing metabolic efficiency [29].4.Hyperinsulinemia suppresses nitric oxide (NO) synthesis via the stimulation of serum- and glucocorticoid-regulated kinase 1, impairing endothelial-dependent vasodilation [31].5.Prothrombotic states develop through augmented platelet clustering and heightened plasminogen activator inhibitor-1 levels, increasing atherosclerotic plaque vulnerability [32].

While the TyG index provides a practical insulin resistance measure through triglyceride and fasting glucose values, its clinical utility is constrained by inherent limitations: susceptibility to dietary influences, age-related variability, and exclusion of key metabolic determinants. In contrast, the eGDR formula integrates waist circumference, hypertension status, and HbA1c—three parameters mechanistically linked to visceral adiposity, chronic inflammation, and glucolipotoxicity [31]. This multi-domain assessment captures synergistic pathological pathways driving endothelial dysfunction and atherosclerosis, including pro-inflammatory cytokine overproduction, oxidative stress amplification, and arterial stiffness progression [33]. Comparative analyses demonstrate eGDR’s superior predictive capacity for cardio-cerebrovascular disease outcomes compared to TyG index alone, particularly regarding stroke risk stratification (AUC (area under the curve) improvement 0.12–0.15, *p*< 0.05) [14,28], likely attributable to its incorporation of obesity-related vascular remodeling mechanisms.

The complementary nature of TyG index and eGDR arises from their distinct pathophysiological insights. While the TyG index captures hepatic insulin resistance through fasting glucose and reflects skeletal muscle/adipose tissue dysfunction via triglycerides [34–35], the eGDR incorporates central obesity metrics (waist circumference), hypertension status, and chronic glycemic burden (HbA1c) – all hallmarks of visceral adiposity-driven insulin resistance [36]. This dual assessment framework addresses synergistic cardiometabolic risks: central obesity exacerbates insulin resistance through adipokine dysregulation, while hypertension and insulin resistance reciprocally amplify endothelial dysfunction and vascular remodeling [37]. By integrating hepatic, peripheral, and adipose tissue-specific insulin resistance markers with obesity-related hemodynamic stressors, the TyG/eGDR combination provides a multidimensional risk profile that surpasses individual parameter limitations, enhancing cardio-cerebrovascular disease prediction through comprehensive metabolic-vascular pathway evaluation.

We observed an antagonistic interaction between the TyG index and eGDR on the multiplicative scale (interaction term OR: 0.76, 95%CI: 0.60–0.95; *p*< 0.05), indicating that the incremental effect of higher TyG on CCVD risk is attenuated when eGDR is already low (i.e., more severe systemic insulin resistance). This finding should not be interpreted as a protective effect. Rather, it likely reflects: (i) a ceiling/saturation phenomenon, whereby key downstream pathways (endothelial dysfunction, oxidative stress, inflammation, thrombosis) are already highly activated at low eGDR, leaving less room for additional multiplicative risk from TyG; (ii) overlapping but non-identical biology, with TyG indexing predominantly hepatic/adipose insulin resistance (very-low-density lipoprotein (VLDL)-TG overproduction, lipolysis) and eGDR capturing peripheral insulin sensitivity and central adiposity (waist circumference, hypertension, HbA1c), so part of the causal pathway is already accounted for when eGDR is low; and (iii) measurement-domain differences/statistical scale issues, whereby antagonism on the multiplicative scale can coexist with meaningful additive risk and high absolute risk in the high-TyG/low-eGDR phenotype. These explanations are also more consistent with our largely non-diabetic cohort (normal HbA1c), making β-cell exhaustion an unlikely driver [38–40].

## 5. The merits and constraints of this research

Merits of this research:1. This investigation represents the first comprehensive evaluation of the combined TyG index and eGDR for predicting cardio-cerebrovascular disease, offering novel insights into integrated metabolic-cardio-cerebrovascular disease risk stratification. 2. The analysis leverages longitudinal data from a nationally representative Chinese cohort with extended follow-up (2011–2020), enhancing the robustness of temporal associations. Nevertheless, the current investigation is not without drawbacks. To begin with, Dynamic fluctuations in TyG and eGDR over time were not analyzed, potentially underestimating their cumulative prognostic impact; second, Subgroup stratification by comorbidities (e.g., hypertension, diabetes) was not performed, limiting insights into disease-specific risk modulation; Third, Generalizability requires validation in multiethnic populations, as findings derive exclusively from the CHARLS cohort, which may not fully represent global demographic and socioeconomic contexts.

The TyG index and eGDR demonstrate strong associations with cardio-cerebrovascular disease, where elevated TyG and reduced eGDR levels independently elevate cardio-cerebrovascular disease risk. Combined assessment of these indices enables refined risk stratification based on insulin resistance severity, enhancing cardio-cerebrovascular disease prediction accuracy. However, their antagonistic interaction suggests complex pathophysiological interdependencies, potentially mediated by compensatory metabolic adaptations in chronic insulin resistance states. These insights highlight the necessity of assessing multi-index insulin resistance profiles to optimize high-risk population identification. Such an approach could inform personalized prevention strategies, advancing precision medicine frameworks for early cardio-cerebrovascular disease intervention.

## Supporting information

S1 File Raw data. (XLSX)

## Abbreviations

AUC: area under the curve
AGE: advanced glycation end-product
AP: attributable proportion
BMI: body mass index
BUN: blood urea nitrogen
CAPI: computer-assisted personal interview
CCVD: cardio-cerebrovascular disease
CHARLS: China Health and Retirement Longitudinal Study
CHD: coronary heart disease
CI: confidence interval
Cox: Cox proportional hazards model
eGDR: estimated glucose disposal rate
FBG: fasting plasma glucose
HbA1c: glycated hemoglobin
HEC: hyperinsulinemic–euglycemic clamp
HDL-C: high-density lipoprotein cholesterol
HR: hazard ratio
IDL: intermediate-density lipoprotein
IR: insulin resistance
IRB: Institutional Review Board
KM: Kaplan–Meier
LDL-C: low-density lipoprotein cholesterol
NO: nitric oxide
OR: odds ratio
PLT: platelet count
RERI: relative excess risk due to interaction
SCR: serum creatinine
SI: synergy index
TC: total cholesterol
TG: triglycerides
TyG: triglyceride–glucose index
UA: uric acid
VLDL: very-low-density lipoprotein

## References

[1] Zhu H, Xie W, Wang P, Jiang S, Hua Y, Shao G, et al. The relationship between blood urea nitrogen to serum albumin ratio and cardiovascular diseases, cardiovascular mortality, and all-cause mortality in patients with diabetes mellitus. Front Endocrinol (Lausanne). 2025;16:1456731. doi: 10.3389/fendo.2025.1456731 40290308 PMC12021637

[2] Li X, Zhao C, Liu M, Zhao W, Pan H, Wang D. Sociodemographic index-age differences in the global prevalence of cardiovascular diseases, 1990-2019: a population-based study. Arch Public Health. 2025;83(1):2. doi: 10.1186/s13690-024-01454-7 39780273 PMC11715713

[3] Center For Cardiovascular Diseases The Writing Committee Of The Report On Cardiovascular Health And Diseases In China N. Report on Cardiovascular Health and Diseases in China 2023: An Updated Summary. Biomed Environ Sci. 2024;37(9):949–92. doi: 10.3967/bes2024.162 39401992

[4] National Health and Wellness Commission of the People’s Republic of China. China Health Statistics Yearbook 2022. Beijing, China: Peking Union Medical College of China Press. 2024.

[5] Zhang W, Song M, Fang Z, Chen F, Yuan H, Gao X, et al. Role of extracellular vesicles in insulin resistance: Signaling pathways, bioactive substances, miRNAs, and therapeutic potential. Cell Biochem Funct. 2024;42(3):e4013. doi: 10.1002/cbf.4013 38639198

[6] Horton WB, Love KM, Gregory JM, Liu Z, Barrett EJ. Metabolic and vascular insulin resistance: partners in the pathogenesis of cardiovascular disease in diabetes. Am J Physiol Heart Circ Physiol. 2025;328(6):H1218–36. doi: 10.1152/ajpheart.00826.2024 40257392 PMC12172477

[7] Feng Y, Yin L, Huang H, Hu Y, Lin S. Assessing the impact of insulin resistance trajectories on cardiovascular disease risk using longitudinal targeted maximum likelihood estimation. Cardiovasc Diabetol. 2025;24(1):112. doi: 10.1186/s12933-025-02651-6 40065358 PMC11895167

[8] Kwak J, Han K-D, Lee EY, Lee S-H, Lim D-J, Kwon H-S, et al. Association between the triglyceride-glucose index and cardiovascular risk and mortality across different diabetes durations: a nationwide cohort study. Endocrinol Metab (Seoul). 2025;40(4):548–60. doi: 10.3803/EnM.2024.2205 40040387 PMC12409153

[9] DeFronzo RA, Tobin JD, Andres R. Glucose clamp technique: a method for quantifying insulin secretion and resistance. Am J Physiol. 1979;237(3):E214-23. doi: 10.1152/ajpendo.1979.237.3.E214 382871

[10] Ryu HE, Lee YJ, Park B, Jung DH. Comparisons of three novel markers for insulin resistance to predict incident cardiovascular disease: a Korean cohort study from three different regions. Eur J Med Res. 2025;30(1):188. doi: 10.1186/s40001-025-02374-0 40114229 PMC11924704

[11] Mansoori A, Allahyari M, Mirvahabi MS, Tanbakuchi D, Ghoflchi S, Derakhshan-Nezhad E, et al. Predictive properties of novel anthropometric and biochemical indexes for prediction of cardiovascular risk. Diabetol Metab Syndr. 2024;16(1):304. doi: 10.1186/s13098-024-01516-4 39696688 PMC11657368

[12] Zheng X, Han W, Li Y, Jiang M, Ren X, Yang P, et al. Changes in the estimated glucose disposal rate and incident cardiovascular disease: two large prospective cohorts in Europe and Asia. Cardiovasc Diabetol. 2024;23(1):403. doi: 10.1186/s12933-024-02485-8 39511639 PMC11545867

[13] Zhao Y, Hu Y, Smith JP, Strauss J, Yang G. Cohort profile: the China health and retirement longitudinal study (CHARLS). Int J Epidemiol. 2014;43(1):61–8. doi: 10.1093/ije/dys203 23243115 PMC3937970

[14] Zhang Z, Tan L. Association of insulin resistance-related indicators with cardiovascular disease in Chinese people with different glycemic states. Front Endocrinol (Lausanne). 2025;16:1515559. doi: 10.3389/fendo.2025.1515559 40313486 PMC12043448

[15] Huang Q, Jiang Z, Shi B, Meng J, Shu L, Hu F, et al. Characterisation of cardiovascular disease (CVD) incidence and machine learning risk prediction in middle-aged and elderly populations: data from the China health and retirement longitudinal study (CHARLS). BMC Public Health. 2025;25(1):518. doi: 10.1186/s12889-025-21609-7 39920658 PMC11806717

[16] Liu X, Zhang H, Li H, Xue F. Cardiovascular-kidney-metabolic syndrome modifies smoking-related risk for cardiovascular diseases: findings from an observational cohort study in UK Biobank. BMC Public Health. 2025;25(1):1609. doi: 10.1186/s12889-025-22865-3 40312716 PMC12044822

[17] Guerrero-Romero F, Simental-Mendía LE, González-Ortiz M, Martínez-Abundis E, Ramos-Zavala MG, Hernández-González SO, et al. The product of triglycerides and glucose, a simple measure of insulin sensitivity. Comparison with the euglycemic-hyperinsulinemic clamp. J Clin Endocrinol Metab. 2010;95(7):3347–51. doi: 10.1210/jc.2010-0288 20484475

[18] Wang Z, Zhu J, Xuan S, Dong S, Shen Z, Chen S, et al. Associations of estimated glucose disposal rate with frailty progression: results from two prospective cohorts. Cardiovasc Diabetol. 2025;24(1):81. doi: 10.1186/s12933-025-02650-7 39972476 PMC11841016

[19] Liang D, Liu C, Wang Y. The association between triglyceride-glucose index and the likelihood of cardiovascular disease in the U.S. population of older adults aged ≥ 60 years: a population-based study. Cardiovasc Diabetol (2024) 23:151. doi: 10.1186/s12933-024-02248-5 38702717 PMC11067197

[20] Liu C, Liang D. The association between the triglyceride-glucose index and the risk of cardiovascular disease in US population aged ≤ 65 years with prediabetes or diabetes: a population-based study. Cardiovasc Diabetol (2024) 23:168. doi: 10.1186/s12933-024-02261-8 38741118 PMC11092030

[21] Rafiee H, Mohammadifard N, Nouri F, Alavi Tabatabaei G, Najafian J, Sadeghi M, et al. Association of triglyceride glucose index with cardiovascular events: insights from the isfahan cohort study (ICS). Eur J Med Res. 2024;29(1):135. doi: 10.1186/s40001-024-01728-4 38368388 PMC10874543

[22] Wang B, Li L, Tang Y, Ran X. Joint association of triglyceride glucose index (TyG) and body roundness index (BRI) with stroke incidence: a national cohort study. Cardiovasc Diabetol. 2025;24(1):164. doi: 10.1186/s12933-025-02724-6 40241070 PMC12004739

[23] Zhang J, Sun Z, Li Y, Yang Y, Liu W, Huang M, et al. Association between the cumulative estimated glucose disposal rate and incident cardiovascular disease in individuals over the age of 50 years and without diabetes: data from two large cohorts in China and the United States. Cardiovasc Diabetol. 2025;24(1):51. doi: 10.1186/s12933-025-02575-1 39891229 PMC11786493

[24] Guo L, Zhang J, An R, Wang W, Fen J, Wu Y, et al. The role of estimated glucose disposal rate in predicting cardiovascular risk among general and diabetes mellitus population: a systematic review and meta-analysis. BMC Med. 2025;23(1):234. doi: 10.1186/s12916-025-04064-4 40264086 PMC12016375

[25] Cui C, Liu L, Zhang T, Fang L, Mo Z, Qi Y, et al. Triglyceride-glucose index, renal function and cardiovascular disease: a national cohort study. Cardiovasc Diabetol. 2023;22(1):325. doi: 10.1186/s12933-023-02055-4 38017519 PMC10685637

[26] Cui C, Liu L, Qi Y, Han N, Xu H, Wang Z, et al. Joint association of TyG index and high sensitivity C-reactive protein with cardiovascular disease: a national cohort study. Cardiovasc Diabetol. 2024;23(1):156. doi: 10.1186/s12933-024-02244-9 38715129 PMC11077847

[27] He H-M, Xie Y-Y, Chen Q, Li Y-K, Li X-X, Mu Y-K, et al. The additive effect of the triglyceride-glucose index and estimated glucose disposal rate on long-term mortality among individuals with and without diabetes: a population-based study. Cardiovasc Diabetol. 2024;23(1):307. doi: 10.1186/s12933-024-02396-8 39175051 PMC11342524

[28] Li Y, Li H, Chen X, Liang X. Association between various insulin resistance indices and cardiovascular disease in middle-aged and elderly individuals: evidence from two prospectives nationwide cohort surveys. Front Endocrinol (Lausanne). 2024;15:1483468. doi: 10.3389/fendo.2024.1483468 39649228 PMC11620891

[29] Hill MA, Yang Y, Zhang L, Sun Z, Jia G, Parrish AR, et al. Insulin resistance, cardiovascular stiffening and cardiovascular disease. Metabolism. 2021;119:154766. doi: 10.1016/j.metabol.2021.154766 33766485

[30] da Silva AA, do Carmo JM, Li X, Wang Z, Mouton AJ, Hall JE. Role of Hyperinsulinemia and Insulin Resistance in Hypertension: Metabolic Syndrome Revisited. Can J Cardiol. 2020;36(5):671–82. doi: 10.1016/j.cjca.2020.02.066 32389340 PMC7219403

[31] Zhang Z, Zhao L, Lu Y, Xiao Y, Zhou X. Insulin resistance assessed by estimated glucose disposal rate and risk of incident cardiovascular diseases among individuals without diabetes: findings from a nationwide, population based, prospective cohort study. Cardiovasc Diabetol. 2024;23(1):194. doi: 10.1186/s12933-024-02256-5 38844981 PMC11157942

[32] Kelem A, Adane T, Shiferaw E. Insulin resistance-induced platelet hyperactivity and a potential biomarker role of platelet parameters: a narrative review. Diabetes Metab Syndr Obes. 2023;16:2843–53. doi: 10.2147/DMSO.S425469 37744701 PMC10516192

[33] Ormazabal V, Nair S, Elfeky O, Aguayo C, Salomon C, Zuñiga FA. Association between insulin resistance and the development of cardiovascular disease. Cardiovasc Diabetol. 2018;17(1):122. doi: 10.1186/s12933-018-0762-4 30170598 PMC6119242

[34] Defronzo RA. Banting Lecture. From the triumvirate to the ominous octet: a new paradigm for the treatment of type 2 diabetes mellitus. Diabetes. 2009;58(4):773–95. doi: 10.2337/db09-9028 19336687 PMC2661582

[35] Lee W-H, Najjar SM, Kahn CR, Hinds TD Jr. Hepatic insulin receptor: new views on the mechanisms of liver disease. Metabolism. 2023;145:155607. doi: 10.1016/j.metabol.2023.155607 37271372 PMC10330768

[36] Actis Dato V, Lange S, Cho Y. Metabolic flexibility of the heart: the role of fatty acid metabolism in health, heart failure, and cardiometabolic diseases. Int J Mol Sci. 2024;25(2):1211. doi: 10.3390/ijms25021211 38279217 PMC10816475

[37] Stanciu S, Rusu E, Miricescu D, Radu AC, Axinia B, Vrabie AM, et al. Links between metabolic syndrome and hypertension: the relationship with the current antidiabetic drugs. Metabolites. 2023;13(1):87. doi: 10.3390/metabo13010087 36677012 PMC9863091

[38] Vasques ACJ, Novaes FS, de Oliveira M da S, Souza JRM, Yamanaka A, Pareja JC, et al. TyG index performs better than HOMA in a Brazilian population: a hyperglycemic clamp validated study. Diabetes Res Clin Pract. 2011;93(3):e98–100. doi: 10.1016/j.diabres.2011.05.030 21665314

[39] Singh B, Saxena A. Surrogate markers of insulin resistance: a review. World J Diabetes. 2010;1(2):36–47. doi: 10.4239/wjd.v1.i2.36 21537426 PMC3083884

[40] Bello-Chavolla OY, Almeda-Valdes P, Gomez-Velasco D, Viveros-Ruiz T, Cruz-Bautista I, Romo-Romo A, et al. METS-IR, a novel score to evaluate insulin sensitivity, is predictive of visceral adiposity and incident type 2 diabetes. Eur J Endocrinol. 2018;178(5):533–44. doi: 10.1530/EJE-17-0883 29535168

